# Revealing spatiotemporal variations in areas potentially linked to COVID-19 spread using fine-grained population data

**DOI:** 10.1038/s41598-025-06658-7

**Published:** 2025-07-02

**Authors:** Nobumasa Ishida, Masashi Toyoda, Kazutoshi Umemoto, Koji Zettsu

**Affiliations:** 1 Department of Information and Communication Engineering, Graduate School of Information Science and Technology, The University of Tokyo, 7-3-1 Hongo, Bunkyo-ku, Tokyo, 1138656 Japan; 2 Institute of Industrial Science, The University of Tokyo, 4-6-1 Komaba, Meguro-ku, Tokyo, 1538505 Japan; 3 Universal Communication Research Institute, National Institute of Information and Communications Technology, 3-5 Hikaridai, Seika-cho, Kyoto, 6190289 Japan

**Keywords:** Infectious diseases, Computer science, Information technology, Software, Statistics

## Abstract

The COVID-19 pandemic has highlighted the need to better understand the dynamics of disease spread in cities in order to develop efficient and effective epidemiological strategies. In this study, we utilise fine-grained spatiotemporal population data obtained from mobile devices to identify areas and time of day that may contribute to COVID-19 spread, and investigate how they change throughout different waves of the pandemic. To evaluate the potential risk to city residents, we analyse the correlation between the effective reproduction number and population dynamics at locations regularly visited by these residents. Our case study of Tokyo identifies highly-correlated areas at a fine-grained level, revealing shifts in these areas within cities and across urban and suburban regions as the pandemic progresses. We also explore the characteristics of the potential areas of concern through the lenses of points of interest and population dynamics. Our findings have implications for comprehensively understanding the spatiotemporal dynamics of COVID-19 and offer insights into public health interventions for managing pandemics.

## Introduction

Emerging in late 2019, the COVID-19 pandemic affected many individuals and placed immense strain on healthcare systems worldwide^1^. A better understanding of the dynamics behind how COVID-19 spreads remains essential for facilitating the development of efficient and effective epidemiological strategies for pandemics, including targeted non-pharmaceutical interventions^2–4^. In this context, substantial efforts have been directed towards identifying infectious hotspots and risky behaviours that could lead to superspreading events. However, the overwhelming number of confirmed cases imposed severe limitations on methods that depend on contact tracing^5–8^. Furthermore, the constantly evolving landscape of the pandemic, characterized by significant changes in human mobility patterns^9,10^ and the emergence of novel viral variants^11^, made tracking the disease increasingly challenging.

Leveraging mobility data collected from mobile devices has emerged as a promising approach to these challenges in the digital age^12–18^. Mobility data serves as a proxy for human mobility patterns, a key driver of the pandemic, and offers the advantage of extensive spatial coverage. Many studies have demonstrated the great potential of mobility data by analysing the association between mobility patterns and the spread of COVID-19^19–21^. One line of research focuses on the heterogeneity of infectious risk through the lenses of different types of activities, points of interest (POIs), and demographic factors^22–29^. Another line focuses on temporal variations regarding these associations throughout successive waves of the pandemic^30–34^. Taken together, these findings highlight multifaceted and evolving risk dynamics in an urban context, posing a persistent challenge for local administrators in determining which areas demand attention at any given time.

In this study, we introduce an approach to systematically identify areas potentially linked to the spread of infection using fine-grained spatiotemporal population data obtained from mobile phones, offering comprehensive coverage across cities. Our method highlights areas of potential concern for city administrators by identifying locations where local population time series tend to synchronise with the effective reproduction number ($R_t$) of each residential city, measured through correlation coefficients. The population dataset provides fine-grained and extensive estimation, consisting of hourly population fluctuation data for individual 125-metre square grids, estimated based on the number of mobile phones detected in each grid. The dataset also includes the distribution of individuals’ residential cities within each grid, which can help reflect variations in $R_t$ across different cities. Rather than directly inferring causal relationships, we use correlation as a screening indicator, with the validity of this approach supported by consistency with findings from prior studies. Our comprehensive investigation reveals spatiotemporal shifts in potential areas of concern within and between cities during different phases of the pandemic. Additionally, analyses from various perspectives—such as examining POIs within the identified areas—provide insights into potentially risky behaviours based on location, time of day, and seasonal events.

We apply this approach to a case study of Tokyo, a densely populated metropolis that has recorded the highest number of confirmed cases in Japan. Our key findings are threefold. First, our method identified areas consistent with prior knowledge of infectious risk, but with a high level of resolution. Examples include several downtown districts, areas along railway lines, and seasonal sightseeing spots. Second, an analysis of three pandemic waves in Tokyo revealed spatiotemporal variations experienced in these extracted areas. During the earlier wave, several metropolitan downtown areas were commonly identified as potentially concerning for residents of different wards (with “wards” referring to central districts within Tokyo). However, as the pandemic progressed, these areas shifted according to the home wards of the residents. Lastly, a similar variation was observed at the level of POIs. While restaurants were predominantly associated with the change in $R_t$ during the earlier wave, this association diminished in the subsequent waves, suggesting a wider spread of the virus throughout the city. Overall, these findings demonstrate the broad range of analyses enabled by our method, providing valuable insights into the dynamic nature of infection risk. In particular, this study highlights the importance of accounting for variations in areas of concern at a high spatial resolution over time, guiding the development of more effective and adaptive public health interventions during future pandemics.

## Results

### Correlation-based potential risk calculation

**Fig. 1:**
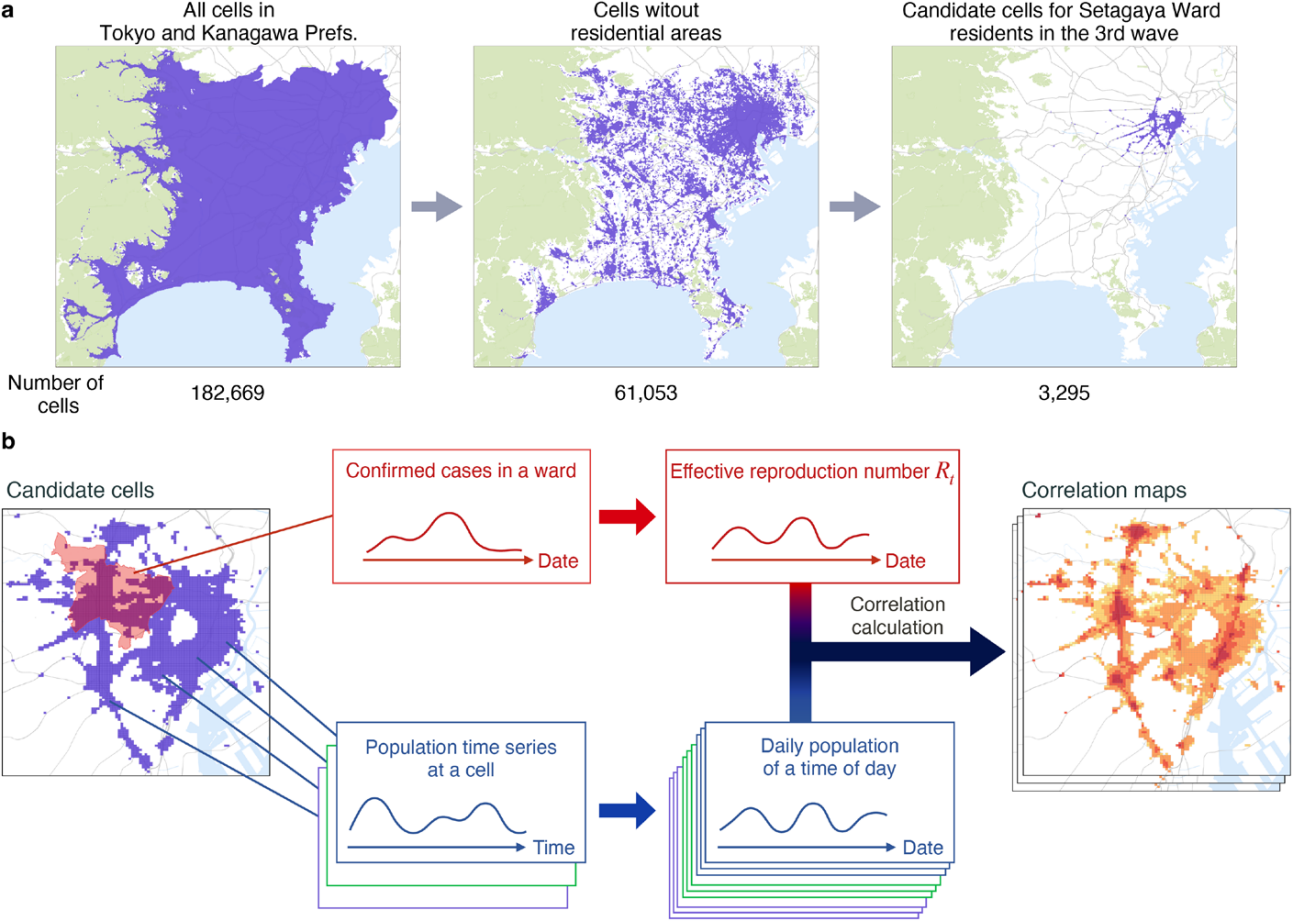
Framework for the potential risk calculation. (**a**) An example of preprocessing in cell screening for Setagaya Ward residents during the third wave. Each cell (125-metre square) is shown in purple. The numbers of extracted cells are shown below the figures. (**b**) The main process for calculating indicators in candidate cells. Our framework assesses the necessity of epidemiological concern by calculating Pearson’s correlation with varying lags between the effective reproduction number of a given ward and the daily population time series of time of day in a given cell. Maps were generated using Kepler.gl (v0.2.2, https://github.com/keplergl/kepler.gl, MIT License), with styles from CARTO (https://github.com/CartoDB/basemap-styles, CC BY 4.0), and tile data from OpenMapTiles (https://openmaptiles.org/, CC BY 4.0) and OpenStreetMap contributors (https://www.openstreetmap.org, ODbL 1.0).

We integrated two datasets for epidemiological modelling. The first was spatiotemporal population data that consisted of hourly population estimates for each 125-metre-square grid cell. The population estimates were grouped by the home wards of the visitors. Provided by LY Corporation (Tokyo, Japan; formerly Yahoo Japan Corporation at the time of data provision), this data was collected from mobile devices of users who had opted in, with privacy-preserving aggregation. It covered 182,669 grid cells across Tokyo and Kanagawa Prefectures, which are central to the Greater Tokyo Area (see the leftmost image in Fig. 1a). Considering the time span of the data, we set the period of our analysis from the third wave (beginning on 2 Nov. 2020) to the fifth wave (ending on 31 Oct. 2021) of the pandemic in Tokyo. The second dataset comprised the daily number of confirmed cases in each ward of Tokyo, aggregated based on their reporting dates. Wards are the smallest units for which confirmed cases were reported in Tokyo.

Our metric of potential risk, or measure of potential concern, was calculated using the most detailed information available from the datasets described above. Specifically, the indicator was defined for each grid cell (*c*) at a given time of day (visiting hour *h*) and was dependent on the residential ward (*w*). The inclusion of ward-specific dependence was intended to capture the varying trends of confirmed cases across wards (see Supplementary Fig. S4) and to provide local administrators with insights into areas requiring heightened attention. The grid cells represented different urban locations (e.g. business or downtown districts), while the time-of-day data reflected distinct types of activities at these locations (e.g. population at 13:00 largely consisting of workers, while population at 20:00 primarily reflecting evening or nightlife activities). We hypothesised that the combination of a specific grid cell and time of day would characterise distinct urban scenarios and activities, which have been shown to influence COVID-19 transmissibility^22^.

Given a triplet (*c*, *h*, *w*), representing these factors, we quantified the potential risk during a wave by calculating Pearson’s correlation coefficient between two daily time series: the daily population at grid cell *c* during hour *h*, denoted as $\textrm{Population}_{d}(c,h)=\textrm{Population}_{t(d,h)}(c)$, and the daily instantaneous effective reproduction number $R_t$ for ward *d*, denoted as $R_d(w)$. Here, $\textrm{Population}_{t}(c)$ is the original hourly time series of population fluctuation in the cell *c*, and *t*(*d*, *h*) represents the time index corresponding to hour *h* on date *d*. Furthermore, to account for fluctuating delays between population changes and subsequent impacts on infection spread (beyond the reporting delays partially corrected in the $R_t$ calculation), we introduced a time shift $\delta$ when calculating correlation coefficients. This was done to mitigate the influence of these potential short-term delays and prevent overlooking potential areas of concern. We applied forward or backward shifts (in days) to the population time series for each cell and calculated the correlation for each shift within a predefined range $[-\Delta , \Delta ]$. The potential risk was then defined as the maximum correlation found across these shifts:

1 $$\begin{aligned} \mathrm{Risk_{\delta }}(c, h, w)&= \textrm{Pearson}[\textrm{Population}_{d+\delta }(c, h), R_d(w)], \end{aligned}$$

2 $$\begin{aligned} \textrm{Risk}(c, h, w)&= \max _{\delta \in [-\Delta , \Delta ]} \mathrm{Risk_{\delta }}(c, h, w), \end{aligned}$$

where the Pearson correlation is calculated over the daily index *d* corresponding to the selected credible period for $R_d(w)$ during the target wave (see “Identifying periods for correlation calculations per ward” subsection), and $\Delta$ is the maximum acceptable lag in days (determined in the “Parameter determination” subsection to be $\Delta =1$).

An overview of our framework is illustrated in Fig. 1b. Here, $R_t$ represents the expected number of individuals infected by a patient at a given time point^35^. We estimated it using a Bayesian method^36^ with data on confirmed cases adjusted for reporting delays (see the “Calculation of *R*_*t*_” subsection of the “Methods” section). Conceptually, our method identifies grid cells where fluctuations in population tend to synchronise with changes in $R_t$  (see the “Risk modelling” subsection of the “Methods” section). While we acknowledge that correlation does not imply causation, previous studies have demonstrated the utility of such correlations in monitoring epidemiological trends^19,30^. Consistent with these findings, we observed in what follows that our approach effectively highlighted potentially concerning areas, aligning with insights from prior research.

To enhance the accuracy of our indicator calculation and address spurious correlations, our method included two steps before computing the indicator for each cell (see corresponding subsections of the “Methods” section). First, we performed a two-phase cell screening process to extract non-residential cells and select candidate cells with more than a certain number of visitors from a target home ward, leveraging home ward information in our spatiotemporal population data. This resulted in 61,053 non-residential cells in Tokyo and Kanagawa Prefectures (about one-third of the total cells), and 3,295 cells for Setagaya Ward during the third wave, for instance. Second, we selected periods with low uncertainty in terms of $R_t$ (i.e. with enough confirmed cases) throughout an entire period of each wave, for correlation calculation. The calibration of the parameters involved and verification are detailed in the “Methods” section.

### Influence of urban-suburban structure on highly-correlated areas

The Greater Tokyo Area comprises a central metropolitan area and suburbs, which are connected to each other mainly by trains. To investigate the impact of this urban-suburban structure on the risk of COVID-19 spread, we analysed correlation maps during the third wave (the initial wave in our datasets) for residents in Setagaya and Shinjuku Wards, as representative suburban and urban wards, respectively. During the wave, Setagaya Ward had the largest number of confirmed cases among all Tokyo’s wards, while Shinjuku Ward had the most cases out of the central wards (see Supplementary Fig. S2).

**Fig. 2:**
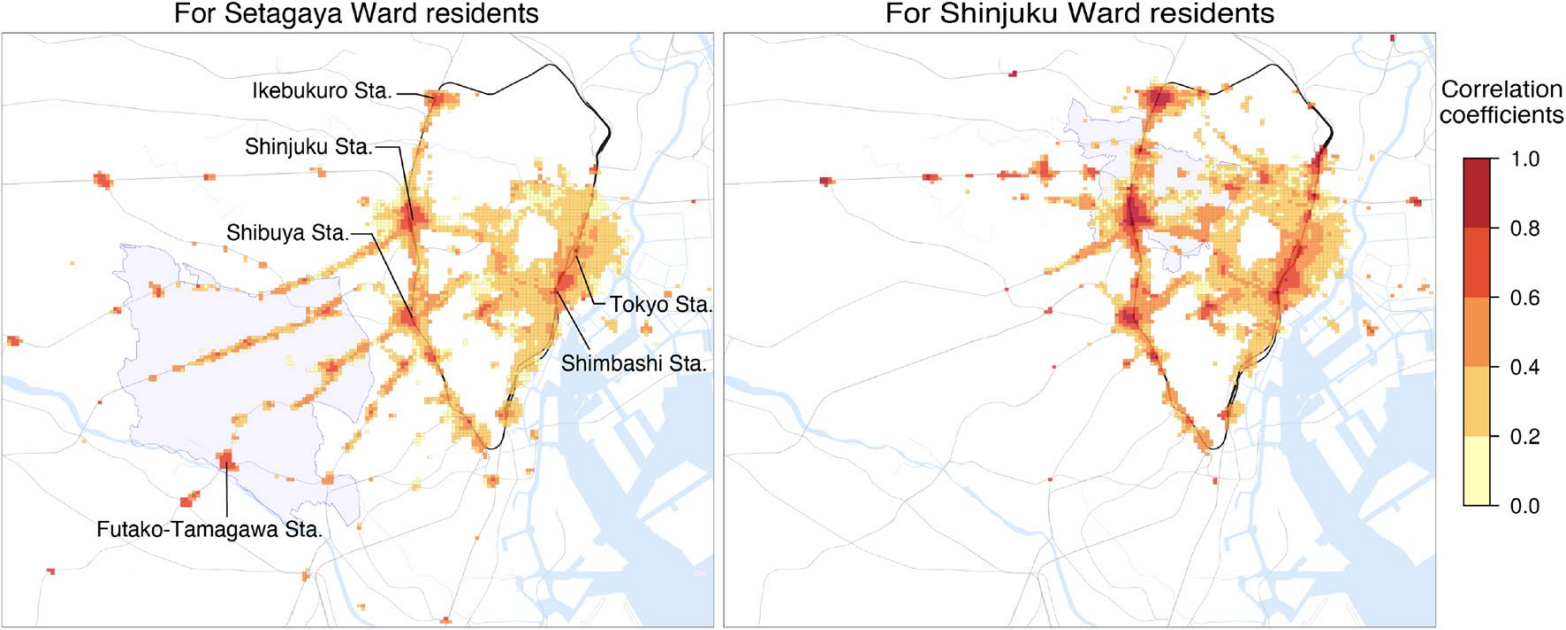
Correlation maps for Setagaya Ward residents and Shinjuku Ward residents in the third wave. The colour of each cell (125-metre square) indicates the highest correlation coefficient at different time of day. The wards for target residents are shown in shades of purple. Railways are indicated by grey curves, with the Yamanote Line highlighted in black. For the sake of visibility, cells with negative correlations or that are far from the metropolitan area have been omitted. Maps were generated using Kepler.gl (v0.2.2, https://github.com/keplergl/kepler.gl, MIT License), with styles from CARTO (https://github.com/CartoDB/basemap-styles, CC BY 4.0), and tile data from OpenMapTiles (https://openmaptiles.org/, CC BY 4.0) and OpenStreetMap contributors (https://www.openstreetmap.org, ODbL 1.0).

The left panel of Fig. 2 shows a correlation map of the third wave for residents in Setagaya Ward. To visualise spatial variation in correlations, each cell in the map is colour-coded based on its highest correlation coefficient among those at all times of day, thus omitting temporal information. From the figure, it can be observed that areas around terminal stations (e.g. Tokyo Sta., Shinjuku Sta., Shibuya Sta., and Ikebukuro Sta.) had high correlation coefficients. This result is consistent with those on infectious risk reported in previous studies^18,26,27,37–39^, validating our metric. The correlation map also highlights areas around railways connected to these stations. We note that the majority of highly-correlated areas were located outside Setagaya Ward, although a few downtown areas within the ward (e.g., around Futako-Tamagawa Sta.) were identified. Additionally, certain areas in neighbouring Kanagawa Prefecture exhibited correlations, albeit rarely (see Supplementary Fig. S6 for a map including Kanagawa).

In the right panel of Fig. 2, we present a correlation map of the third wave for residents in Shinjuku Ward. Once again, areas around the terminal stations showed high correlations. However, in contrast to Setagaya Ward’s map, Shinjuku Ward’s map contained most of the high-correlation areas inside the Yamanote Line, the main circular railway surrounding the central metropolitan area of Tokyo.

### Spatiotemporal variations of potential areas of concern

**Fig. 3:**
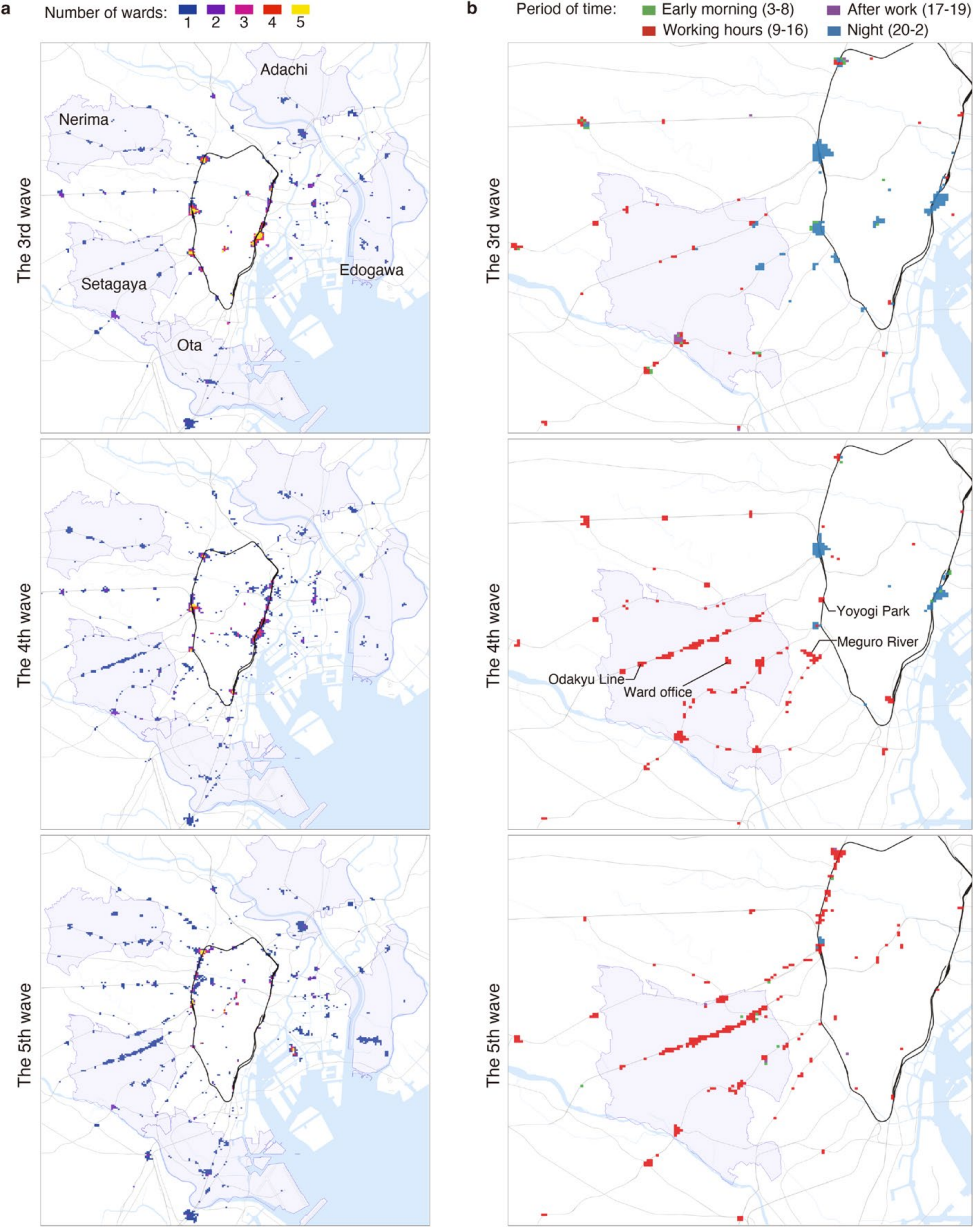
Spatiotemporal variations of cells of potential concern. (**a**) Cells of potential concern extracted for the target five wards. The colour of each cell depicts the number of wards that identify the cell as potentially concerning. The target wards are shown in purple. Railways are indicated by grey curves, with the Yamanote Line highlighted in black. (**b**) Cells of potential concern for Setagaya Ward residents at different waves. Setagaya Ward is shown in purple. The colour of each cell represents the time of day for which the population time series showed the highest correlation with $R_t$. For the sake of visibility, some cells far from the metropolitan area have been omitted. Maps were generated using Kepler.gl (v0.2.2, https://github.com/keplergl/kepler.gl, MIT License), with styles from CARTO (https://github.com/CartoDB/basemap-styles, CC BY 4.0), and tile data from OpenMapTiles (https://openmaptiles.org/, CC BY 4.0) and OpenStreetMap contributors (https://www.openstreetmap.org, ODbL 1.0).

Next, we analysed the spatiotemporal characteristics of the areas requiring heightened attention. We first identified the top 300 cells with high correlation coefficients for each wave and ward as *cells of potential concern*, which accounted for approximately 0.5% of the non-residential cells. Here we emphasise that our method highlights areas requiring further epidemiological consideration, but does not directly quantify the possibility of infection at these areas. To investigate whether these cells of potential concern were common across different wards, we selected Adachi, Edogawa, Nerima, Ota, and Setagaya as target wards (Fig. 3a), which had the largest number of confirmed cases during the third to fifth waves (see Supplementary Fig. S2). We counted the number of wards that shared each cell of potential concern for each wave. As shown in Fig. 3a, cells of potential concern for the third wave were concentrated in the central metropolitan area (through which the Yamanote Line runs) and shared by multiple wards. However, as the pandemic progressed, these cells became more distinct for each ward, and tended to shift to the suburbs.

To better understand the spatiotemporal variations of cells of potential concern over different waves, we next focused our analysis on Setagaya Ward, which consistently had the largest number of confirmed cases. Figure 3b shows these cells for Setagaya Ward residents during each of the three waves. The areas identified for the later waves contained fewer metropolitan areas (including the terminal stations mentioned above) and more areas within Setagaya Ward. For example, cells of potential concern in the fourth wave were located near the ward office and along the Odakyu Line, a railway connecting Kanagawa Prefecture (below Tokyo in the figure) to Shinjuku Station through Setagaya Ward. Other examples included Yoyogi Park and Meguro River, popular spots for viewing cherry blossoms during their blooming period (the end of March until the beginning of April). For the fifth wave, the cells of potential concern were mainly located near railway lines. These results imply a possible association between railway usage and surges in infections, which is in accordance with the findings of previous studies^40,41^.

We also analysed the time of day with a higher correlation. To do this, we classified the time of day into four categories: early morning (3:00–8:59), working hours (9:00–16:59), after work (17:00–19:59), and night (20:00–2:59). Figure 3b shows the time categories for which each cell had the highest correlation with $R_t$. While cells of potential concern for the third wave were concentrated at night, those in the fourth and fifth waves were mostly associated with working hours. In particular, downtown districts around major terminals exhibited a high correlation at night for the third and fourth waves, indicating that off-time activities after working potentially contributed to the spread of COVID-19 during these waves. Conversely, areas including downtowns in suburbs and those surrounding railway lines were highly correlated during working hours for the fourth and fifth waves. This could suggest that daytime activities, including working, were associated with the disease spread during these waves.

### Points-of-interest (POIs) in potential areas of concern

**Fig. 4:**
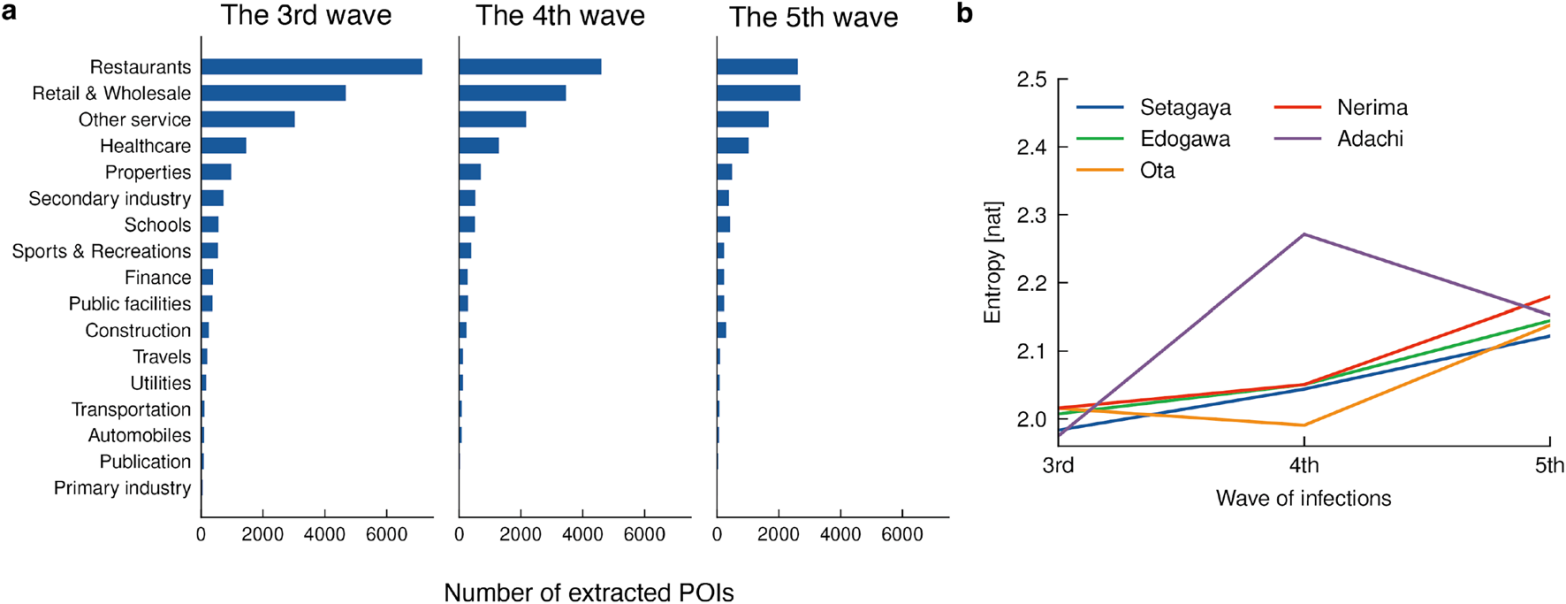
POI statistics on the potential areas of concern. (**a**) The number of POIs included in the cells of potential concern for Setagaya Ward residents. (**b**) Entropy of the POI distributions for each home ward.

The above analysis revealed that areas of potential concern exhibited certain spatiotemporal patterns. To gain a better understanding of the dynamics of these patterns, we analysed the POIs associated with these areas using a POI dataset consisting of 634,107 POIs in Tokyo and Kanagawa prefectures, along with their locations and categories (see the “Points-of-interest (POIs) data” subsection under the “Methods” section). Specifically, we counted the number of POIs within cells of potential concern for each category. Figure 4a shows the distribution of POIs detected for Setagaya Ward residents during each wave. During the third wave, the number of detected POIs varied greatly from category to category; in particular, “Restaurants” was the most common category, which is consistent with previous findings^22,26,38^. Note that this large variance cannot be attributed solely to differences in the total POI counts among the categories (see Supplementary Fig. S9). Subsequently, there was a gradual decrease in this variance over time. We observed the same trend in other wards as well (see Supplementary Figs. S10–S13).

To quantify the temporal changes in variance, we computed the entropy of the POI distribution for the five wards shown above. From Fig. 4b a monotonic increase of entropy was observed for all of the wards except Adachi, for which the maps of potentially concerning areas contained significant noise during the fourth wave (see Supplementary Fig. S7). These results suggest that, as the pandemic progressed, the spread of COVID-19 was no longer attributable to any single POI category, and may have been associated with many different POIs.

### Population time series at potential areas of concern

**Fig. 5:**
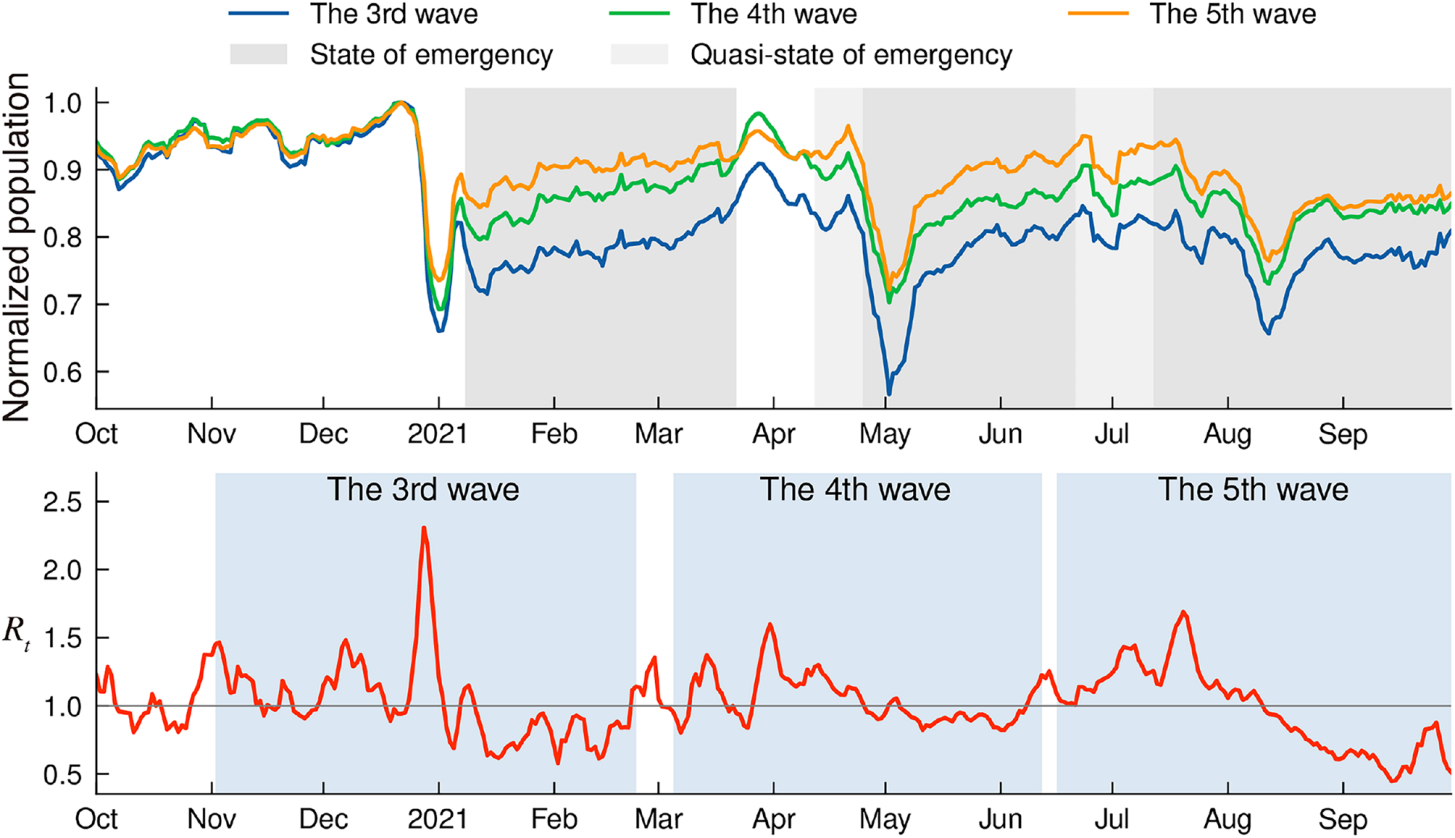
Comparison of populations in potential areas of concern for each wave and $R_t$ time series. Upper row: Average population time series in cells of potential concern at their most highly-correlated time of day, for Setagaya Ward residents. The line labelled “The *N*th wave” was calculated based on the cells of potential concern for the *N*th wave. Each time series was normalised to have a maximum value of 1 during the plotted period. The periods during which the (quasi-)SoEs were declared are depicted with grey shading: details of specific restrictions are described in Supplementary Table S2. Lower row: $R_t$ time series of Setagaya Ward with reporting delays corrected. The periods of waves are depicted in blue shading. For the sake of visualisation, the plot range of the date was cut off on 30 September 2021 to avoid artificial jumps of $R_t$ in October 2021 due to a low number of confirmed cases.

Finally, we analysed the population time series of the highly-correlated areas directly to explicitly investigate the temporal events that caused a high correlation between these time series and $R_t$. Over the entire analysis period, we calculated the average of the population time series at the top 300 cells identified as potentially concerning for each wave, for Setagaya Ward residents. Each cell’s contribution to the average is its population time series evaluated consistently at the specific hour of day ($h_{\max}$) that yielded its highest correlation during wave *N*. The resulting average time series, calculated over the entire study duration shown, is then normalised by dividing by its maximum value observed within the plotted date range to set the displayed peak to 1. The three resulting time series, which reflect the set of cells of potential concern for each wave, are plotted in Fig. 5, juxtaposed with the $R_t$ time series of the ward.

In the figure, the three population time series exhibit similar upward and downward trends on the whole, but there are also some notable differences between them. In what follows, we detail how the population and $R_t$ time series changed for the period of each wave. We note that, during the pandemic, the Tokyo Metropolitan Government declared (quasi-)states of emergency (SoEs) several times, including non-compulsory requests for citizens to refrain from going out^42,43^, which may have affected mobility patterns^44^.

During the third wave (2 Nov. 2020 to 23 Feb. 2021), we observed that the population time series of cells of potential concern of the third wave experienced the largest drop around New Year’s Day among those of the three waves, which coincided with the rapid decline of $R_t$. The sharp peak of $R_t$ before the decline is partially due to the suspension of new case reporting over the New Year holidays. The population remained low compared to these cells of the other waves during the SoE in early 2021. Our POI analysis suggests that the dynamics of populations in potentially concerning areas during the third wave may have been related to year-end parties at restaurants.

During the fourth wave (5 Mar. 2021 to 12 Jun. 2021), both the population and $R_t$ peaked at the end of March, coinciding with the season for the spring vacation and cherry-viewing parties. This trend aligns with our analysis of potential areas of concern discussed in the previous section. A significant drop in the population was then observed around the end of April and the beginning of May, likely attributable to a declared SoE and the national holidays in Japan. By contrast, $R_t$ continued its gradual decline after peaking at the end of March and did not mirror the population’s recovery in mid-May, leading to the abatement of the wave. This divergence between the population and the $R_t$ time series suggests that daily human mobility did not solely dictate $R_t$ trends, particularly toward the end of a wave.

Finally, during the fifth wave (16 Jun. 2021 to 31 Oct. 2021), both the population time series for cells of potential concern and the $R_t$ time series showed a decreasing trend from mid-July to mid-August, which includes consecutive public holidays and a major holiday season in Japan. Later, while the population time series increased, the $R_t$ time series continued to decrease. This discrepancy may have been due to the increased spread of vaccination among the population with Pfizer/BioNTech and Moderna vaccines^45,46^ by that time (see Supplementary Fig. S5 for statistics of the vaccination ratio in Japan^47,48^).

## Discussion

We proposed a fine-grained potential risk analysis framework for COVID-19 that calculates correlations between populations and $R_t$ time series. We presented our case study of Tokyo where we analysed potential areas of concern from the following four perspectives: urban-suburban structure, spatiotemporal variations, POIs, and population time series. Our results showed that the metropolitan centres were potentially concerning at night during the third wave of infection, which is consistent with that noted in several previous reports discussed below. Our analyses across multiple waves and wards revealed that potential areas of concern moved from metropolitan centres to suburbs for the latter waves. Therefore, our results not only agree with previous findings regarding the characteristics of high-risk areas, but also provide new insights into the dynamics of COVID-19 infection throughout Tokyo, with an unprecedented level of resolution. This case study demonstrates the abundant implications provided by our approach.

As regards the third wave, we showed that areas around major terminal stations, where many restaurants and bars are located, were potentially risky at night. This result aligns with the findings of a previous study from the United States^22^ and the discussion at the COVID-19 monitoring meeting held by the Tokyo Metropolitan Government^38^. Our analysis of the population time series also indicated that the populations in those areas rapidly decreased at the end of 2020, along with the corresponding $R_t$ time series. These results suggest that drinking and eating activities at year-end and New Year parties may have driven the third wave of infection.

As regards the fourth wave, our findings indicate a significant shift in the distribution of potentially concerning areas, moving from common metropolitan regions at night to localised areas within individual home ward during working hours. This transition is possibly attributable to a rise in workplace infections, representing a departure from the previous wave’s focus on restaurant-related cases. Corroborating this observation, the Tokyo Metropolitan Government reported a significant increase in workplace-related infections^49^. Our findings on the potential risk of the disease spread within and outside individual home wards in Tokyo offer new perspectives that complement the report.

As regards the fifth wave, our analysis of the population time series showed that the correlation between the population time series of potential areas of concern and $R_t$ weakened in the latter part of the wave. This trend might be due to the spread of vaccination, as evidenced by the rapid increase in the population considered fully-vaccinated (see Supplementary Fig. S5). However, as was shown in our analysis of spatiotemporal variations and POIs, potentially concerning areas remained identifiable as regions that provided insights into the dynamics of COVID-19 infection. We attribute this success to the duration of the period used for our indicator calculation, which appears to be sufficient for capturing the correlation between the population and the $R_t$ time series in the earlier part of the wave. We note that the Tokyo 2020 Olympic Games were held during the fifth wave, but areas directly related to the game were not analysed.

This study reveals the spatiotemporal variations of potential areas of concern at a high resolution of 125-metre squares. Identifying these areas at this level of resolution may help governments update their targets for fixed-point monitoring of areas of concern in a more adaptive and effective manner^38^. Furthermore, our analysis, particularly the comparison of population dynamics across waves (Fig. 5), offers insights related to interventions like the SoEs. The population time series for areas associated with the third wave (predominantly central, nighttime locations) showed a marked decrease during the first SoE in early 2021 and remained relatively suppressed compared to pre-SoE levels. This suggests interventions targeting these settings impacted activity there^50^. However, the observed shift towards more suburban, daytime locations in the fourth and fifth waves (Fig. 3), coupled with population dynamics showing less drastic reductions during later SoEs in these areas (Fig. 5), suggests an evolving situation. While interventions might have reduced risk in initial central hotspots, transmission likely shifted towards activities and locations less affected by restrictions or where behaviour adapted. The growing diversity of POIs associated with risk areas (Fig. 4b) supports this notion. This highlights the challenge for public health authorities to adapt monitoring and interventions as high-risk settings change throughout a prolonged pandemic.

Since our method is primarily based on population data collected from mobile phones and the inferred $R_t$ time series, it is important to assess the accuracy of these data sources as representations of real-world conditions. First, population data can suffer from sampling biases owing to discrepancies between recorded statistics regarding mobile phone users and actual population characteristics. As we applied temporal smoothing to the population time series (see the “Spatiotemporal population data” subsection of the “Methods” section) and calculated all correlation coefficients using data from time periods that exceeded 1 month, we consider that the potential effect of the sampling bias was partially alleviated. In addition, the population data collected by LY Corporation has already been used in previous studies on COVID-19^18,37,44^, supporting its applicability for this analysis.

Second, $R_t$ time series can be distorted if the testing capacity is reached. To mitigate this, we ensured that there was excess testing capacity across Tokyo over all of the periods we analysed. The Tokyo Metropolitan Government announced their plan to enable the conduct of 97,000 COVID-19 tests per day in April 2021, and the maximum number of tests per day in Tokyo until the end of the fifth wave was 25,678^51,52^.

Third, the uncertainty of $R_t$ can be large if the number of confirmed cases is small. In our method, roughly $\ge$ 18 cases per day on average were required to calculate $R_t$ reliably (see the “Parameter determination” subsection of the “Methods” section). As we used the number of cases recorded by ward, the number of cases per day was much larger than the minimum required for a reliable analysis during the selected waves.

Fourth, our $R_t$ estimates are based on total reported cases per ward, which inevitably include household transmissions, while our population analysis focuses on non-residential locations to capture community transmission dynamics. However, its impact may be moderated because a significant portion of cases reported in Tokyo during the study period had unknown transmission routes (often exceeding 60%^53^), and even among cases with known routes where household transmission was frequent (e.g., accounting for 68.9% of known-route cases in July 2021^54^), this often follows initial community acquisition, suggesting household transmission accounted for roughly a third ($\sim$28%) of total cases during such periods. Therefore, fluctuations in community transmission, driven by mobility patterns in the areas we study, are still expected to be a major driver of the overall $R_t$ trend. Nevertheless, the inability to isolate community-acquired cases for $R_t$ calculation remains a limitation inherent to using publicly reported aggregated case data.

Our study was retrospective and aimed to reveal the spatiotemporal dynamics of potentially concerning areas of Tokyo during the past waves of COVID-19 infection. It is important to note that our method used a practical $R_t$ time series, which inevitably introduced reporting delays that may render the approach unsuitable for capturing current state of infection spread. The requirement of conducting a stable calculation of correlation coefficients over a period of approximately 1 month introduces an additional delay as well. As such, a promising direction for future research would be to conduct real-time analyses to facilitate even more effective implementation of public health interventions. Moreover, while our correlation-based approach serves as a scalable screening tool, future work could explore more complex time-series models, such as Vector Autoregression, for a deeper investigation of the dynamic relationship between mobility and $R_t$ in specific high-interest locations identified by our method, provided computational feasibility and data requirements can be met.

## Methods

### COVID-19 data in Tokyo

We acquired two datasets on confirmed COVID-19 cases in Tokyo from the Tokyo Metropolitan Government^55^ (currently available from ref. 53). The first dataset provided information on the number of confirmed cases per ward. As this data was presented as cumulative confirmed cases in each ward based on reporting day, we computed the daily number of newly confirmed cases per ward by calculating the differences between two consecutive days. The second dataset included details on the date of COVID-19 onset and reporting days in Tokyo. Each entry corresponded to a confirmed case in Tokyo, and provided details about the date of onset and the date of the report, if available.

Vaccination data for Tokyo was sourced from the Digital Agency of the Japanese government^47^ (currently available from the Ministry of Health, Labor and Welfare of Japan^56^). The total population used to calculate the ratio of the vaccinated population was obtained from the Bureau of Social Welfare of the Tokyo Metropolitan Government^48^ (currently available from ref. 57). See Supplementary Fig. S5 for the ratio of the vaccinated population in Tokyo during the period analysed.

### Spatiotemporal population data

The spatiotemporal population data used in this study was obtained from LY Corporation (Tokyo, Japan; formerly Yahoo Japan Corporation at the time of data provision), a leading internet company in Japan. This dataset provided estimates of hourly population counts for each cell, which were grouped by home municipalities and wards. The cells were defined as 125-metre squares according to Japanese Industrial Standards X0410^58^, and covered the Tokyo and Kanagawa prefectures. The data spanned from 1 September 2020 to 30 November 2021. The spatiotemporal population data was based on the location data of users who were using LY Corporation’s smartphone apps and consented to their usage policies. This individual location data underwent preprocessing and filtering to estimate the duration of the user’s stay in each cell, thus mitigating the influence of individuals passing only momentarily through cells. The data also included home city information estimated through a proprietary company method. Before providing the data to the authors, Yahoo Japan Corporation performed anonymisation procedures, such as data aggregation and preprocessing, to ensure privacy. Although the $R_t$ values used for correlation are specific to Tokyo wards, the population data covers both Tokyo and Kanagawa, allowing us to identify potentially relevant locations visited by Tokyo residents in either prefecture.

To reduce noise in the population data, we applied the following preprocessing steps before conducting our potential-risk calculations. First, we averaged the population counts in each cell for the target hour and the neighbouring two hours. We then calculated a 7-day rolling average using the daily population time series for each time of day and each cell, thereby addressing weekly fluctuations.

### Data on facilities with publicly reported confirmed cases

To adjust the parameters in our method, we leveraged the data obtained from JX PRESS Corporation (Tokyo, Japan), which specifically pertained to facilities where visitors or staff members tested positive for COVID-19. These facilities were identified through publicly available data sources such as administrators’ or companies’ websites. Each entry in the dataset contained the facility’s location, the date of the initial report, the latest update, and the number of reported cases. The facilities included businesses such as restaurants, retail stores and offices.

While the data included counts of reported cases per facility, we opted to treat all facilities equally without considering the numerical value. This was because the data from some of the facilities was not consistently updated following the modification of source entries. To focus on community-acquired infections, we excluded facilities whose names contained Japanese words associated with hospitals, nursing homes, schools, and kindergartens (see Supplementary Table S4).

### Points-of-interest (POIs) data

Data regarding POIs in Tokyo and Kanagawa prefectures was obtained from phone book data provided by Nippon Software Service Corporation (Tokyo, Japan), which was last updated in 2020. Each entry in the dataset included the POI’s name, latitude, longitude, and an associated category (e.g., Restaurants, Retail & Wholesale, Healthcare, Finance [e.g., banks, securities firms], Travels [e.g., hotels, travel agencies], Properties [e.g., real estate offices], Publication [e.g., publishing houses, bookstores]). To ensure that there were no duplicate entries for POIs with multiple phone numbers, we considered entries to represent a single POI if they had the same position and the first four letters of their names were identical. As a result, we identified a total of 634,107 unique POIs. We also omitted any POIs that did not have category information available. Therefore, the final dataset contained 599,708 POIs.

### Calculation of $R_t$

We calculated $R_t$ for each ward using reported case data. This calculation was performed via the following two steps. First, we estimated confirmed cases based on infection day by adjusting the reported cases to account for delays from the date of infection until the date they were officially reported. The delay from infection to onset was assumed to be 5.6 days, as has been previously reported^59^. We also calculated the mean delay from onset to report using detailed patient information in Tokyo: 5.4 days for the third wave (number of patients included: $n=48,976$ and 95% Confidence Interval (CI): 5.35–5.42), and 4.5 days for both the fourth ($n=35,142$ and 95%CI: 4.48–4.56) and fifth waves ($n=158,565$ and 95%CI: 4.51–4.55).

Next, we calculated $R_t$ from the case information based on the infection day by using the Bayesian method described by Thompson *et al.*^36^, implemented in the R package “EpiEstim”. We used a gamma distribution with a mean of 4.8 days and a standard deviation of 2.3 days as the serial interval for COVID-19^60^. Although the various SARS-CoV-2 variants emerged, such as the Alpha (B.1.1.7) variant in the fourth wave and the Delta (B.1.617.2) variant in the fifth wave^61^, we used the same serial interval for all of the waves. In terms of the prior distribution of $R_t$, we used a gamma distribution with a mean of 5 and a standard deviation of 5, which are the default values for the method^36^. To mitigate fluctuations in reported cases within a week, we applied a 7-day window time centred around the specific day when calculating $R_t$ using “EpiEstim”. The calculated $R_t$ values are presented in Supplementary Fig. S4.

### Identification of COVID-19 waves in Tokyo

Over the period leading up to 30 October 2021, Tokyo endured five distinct waves of COVID-19 since the confirmation of the first local cases. To accurately delineate each wave, we adopted the following definition: a wave commenced on the day when the 7-day rolling average of confirmed cases, based on the reporting day, began to rise then concluded on the day when the number of cases stopped declining. The specific time periods corresponding to each wave are presented in Supplementary Table S1 and Supplementary Fig. S1

### Identifying periods for correlation calculations per ward

We identified periods when $R_t$ was credible. Initially, we computed the coefficients of covariance (CVs) for the posterior distribution of $R_t$. We then identified the longest time intervals where the CV remained below a threshold, for each ward and wave. The methodology used for threshold determination is further detailed in the “Parameter determination” subsection. The identified time intervals for each ward are presented in Supplementary Fig. S3 and Supplementary Table S3.

### Extracting non-residential cells

To focus on community-acquired infections and exclude household infections, we used spatiotemporal population data to extract non-residential cells. A non-residential cell was defined as a cell in which the daytime population exceeded the morning population on weekdays. Specifically, we conducted a one-sided Student’s *t*-test to compare the mean population at 6 a.m. during a given period with that at 12 p.m. The 6 a.m. was selected to capture the population at home. Choosing 7 a.m. would have introduced potential bias from commuting dynamics, while 5 a.m. would have resulted in an underestimation of the population because the data was based on devices with corresponding Apps that had to be turned on. Cells with no population data at either 6 a.m. or 12 p.m. during the given period were excluded from the analysis.

We used population data from a period without a state of emergency to extract non-residential cells, in order to mitigate the influence of stay-at-home behaviour on COVID-19. We chose September 2020 for this purpose. During this period, there were 20 weekdays and no declaration of a state of emergency (see Supplementary Table S2)^42^.

The significance level for the t-test was set at $\alpha =0.01$. The extracted cells are presented in Fig. 1a. Out of a total of 182,669 cells in Tokyo and Kanagawa prefectures, we identified 61,053 cells as non-residential. We observed high coverage in central Tokyo and identified other metropolitan areas.

### Screening areas based on the number of population and visitors

To identify areas that had a significant impact on infections of residents in a specific ward, we identified candidate cells that met two criteria: a population exceeding a certain threshold, and a certain frequency of visits. Specifically, we extracted cells with maximum values in terms of 7-day average population and visitor counts from the given ward equal to or greater than certain thresholds. The specific threshold values varied between wards, owing to variations in permanent populations. The “Parameter determination” subsection provides more detailed information regarding how these thresholds were determined.

### Risk modelling

Our approach used the time-varying effective reproduction number $R_t$ to assess the transmissibility of COVID-19 on a day-to-day basis^35^. We approximated $R_t$ as the product of two key parameters: $R_t = C_t P_t$, where $C_t$ represents the average number of effective contacts with susceptible individuals per infected case, and $P_t$ denotes the probability of transmission from an infected to a susceptible individual per effective contact^62^.

To integrate the spatiotemporal population data with $R_t$, we made an approximation that the time series of the number of effective contacts $C_t$ was proportional to the daily time series of the population in a specific area and time of day. We also posited that the temporal fluctuations in $R_t$ were predominantly driven by changes in the average number of effective contacts $C_t$, while transmissibility $P_t$ remained relatively constant within a given infection wave. This assumption was based on our focus on the short-term variability of $C_t$, which was measured on a daily scale and could be considered considerably more dynamic compared to $P_t$.

We used $R_t$ for each ward *w* to account for the heterogeneity observed in the dynamics of confirmed cases per ward. We considered the infectious risks of the residents of the ward *w* within a given cell *c*, over the course of a given *h*. Based on these factors, we assumed that if a population group gathered within *c* at *h* exhibited a large number of infections, then the daily population time series in that group, $\textrm{Population}_d(c,h)=\textrm{Population}_{t(d,h)}(c)$, would correlate with the $R_t$ of the target ward. Thus we defined the potential risk of infection by the establishment of $R_d(w) \propto \textrm{Population}_d(c,h)$ for the group, which was measured by Pearson’s correlation coefficient between the two time series as in Eq. (1). This correlation-based metric serves as a screening indicator of potentially risky areas requiring further epidemiological investigation, rather than a direct inference of causal relations.

In order to account for fluctuating delays between infection and reporting, we introduced a time shift $\delta$ (in days) when calculating correlation coefficients. This was done to mitigate the influence of these delays and prevent oversight in potential areas of concern. While the calculation of $R_t$ partially corrected for these delays, there were still unknown lags between the $R_t$ values and the population time series. To address this issue, we applied forward or backward shifts to the population time series for each cell as in Eq. (2), up to a specified number of days. These shifts were determined based on the lag that yielded the highest correlation coefficient for that particular cell. The “Parameter determination” subsection contains further detailed information regarding specifically how we determined the maximum length of acceptable lags $\Delta$.

### Parameter determination

The proposed method required the following parameters to be determined:

- **Threshold of population** to extract cells with substantial populations.
- **Threshold of the number of visitors** from a target ward to extract relevant cells for that ward.
- **Threshold of CVs of** $R_t$ to select periods with low uncertainty of $R_t$.
- **Maximum acceptable lag (**$\Delta$**)** to mitigate fluctuation of lags between population and $R_t$.

We determined these parameters by optimising the method’s ability to identify locations associated with known COVID-19 occurrences, using data regarding facilities with publicly reported confirmed cases during the third wave (2 Nov. 2020 to 23 Feb. 2021). We focused on the third wave due to the availability of the facility data. In this section, we refer to these facilities as *facilities with infections*. We formally defined these as facilities whose first report date or last update date fell within the third wave period, excluding specific types like hospitals and schools (see “Data on facilities with publicly reported confirmed cases” subsection). We used the Setagaya Ward case during the third wave as the primary basis for parameter tuning, given its high case numbers and suburban characteristics, and then adapted the visitor threshold for other wards based on population ratios.

The parameter determination involved the following steps:

1. **Defining Search Ranges:**
  *Population threshold:* We first identified non-residential cells visited by at least one person from any of the target wards (Adachi, Edogawa, Nerima, Ota, Setagaya, Shinjuku) during the third wave. Then, for each such cell, we calculated its maximum daily population (7-day rolling average, across all hours) during the third wave. A histogram of these maximum population values was constructed. We set the search range for the population threshold from the median (2900) to the 75th percentile (4500) of this distribution. This range focuses on cells with significant populations while retaining potentially relevant suburban locations.
  *Visitor threshold (Setagaya baseline):* We searched for the threshold for the number of visitors from Setagaya Ward in the range of 0–300 (increments of 10). A value of ‘0’ implies any cell with at least one visitor is included.
  *CV threshold for* $R_t$: We searched within the range of 0.05–0.20 (increments of 0.01). We excluded any threshold value that resulted in a selected time period for correlation calculation shorter than 30 days to ensure stability.
  *Maximum acceptable lag * ($\Delta$): We searched $\Delta$ from 0 to 7 days (increments of 1 day). This defines the range $[-\Delta , \Delta ]$ for the shift $\delta$ in Eq. (2).
2. **Evaluation Score Calculation:** For each combination of parameters within the search ranges, we performed the following:
  Filtered cells based on the population and visitor thresholds.
  Identified the time period for correlation calculation based on the CV threshold.
  Calculated the risk metric $\textrm{Risk}(c, h, w)$ for each remaining cell *c*, hour *h*, and the target ward *w* (Setagaya), using the specified maximum lag $\Delta$. For each cell *c*, we identified the hour $h_{\max}$ that yielded the highest risk value, $\textrm{Risk}(c, h_{\max}, w)$.
  Ranked all filtered cells in descending order based on their maximum risk value $\textrm{Risk}(c, h_{\max}, w)$.
  Calculated the cumulative number of unique *facilities with infections* located within the top *k* ranked cells, denoted as *n*(*k*).
  Computed an evaluation score similar to an Area Under the Curve (AUC) but focusing on the top-ranked cells relevant to our later analyses. Specifically, we calculated the sum of *n*(*k*) for $k = 1,..., K$:
    3 $$\begin{aligned} \textrm{AUC} = \frac{1}{K}\sum _{k=1}^{K} n(k), \end{aligned}$$
    where we set $K=300$ in this paper. A higher score indicates better performance in concentrating relevant facilities among the top 300 ranked cells.
3. **Parameter Selection:** We selected the parameter combination that yielded the highest evaluation score. The optimal parameters found for Setagaya Ward (Wave 3) were:
  Population threshold: 4500
  Visitor threshold (Setagaya baseline): 140
  CV threshold: 0.10
  Maximum acceptable lag ($\Delta$): 1 day (meaning shifts $\delta = -1, 0, +1$ were tested)
  The CV threshold of 0.10 corresponds to requiring approximately 18 cases per day on average over a 7-day window for reliable $R_t$ estimation, consistent with previous work^35^. To establish the appropriate threshold of the number of visitors for other wards, we multiplied the value of 140 by the ratio of the total population of each ward to that of Setagaya Ward, using population data from Ref.^63^.
4. **Validation with Baseline:** To validate the effectiveness of using correlation as a ranking criterion, we also calculated the evaluation score using a baseline ranking based solely on the maximum 7-day average population (across all hours) in each cell. The score achieved using the correlation-based ranking (272.107) significantly exceeded the baseline score (217.153), supporting the utility of our risk modelling approach compared to a naive population-based ranking. Moreover, we plot the top 300 cells with respect to these rankings in Supplementary Fig. S8. From the figure, we find that the potential areas of concern by our metric better cover the locations with the facilities with infections.

## Supplementary Information

Supplementary Information.

## Data Availability

The spatiotemporal population data used in this study was provided by LY Corporation (formerly Yahoo Japan Corporation at the time of data provision) and can be purchased through their DS.ANALYSIS service (https://ds.yahoo.co.jp/service/analysis/). The data on facilities with confirmed cases was provided by JX PRESS Corporation and can be purchased through their FASTALERT service (https://fastalert.jp/). The POI data was provided by Nippon Software Service Corporation and can be purchased through their telephone book database service (https://www.nipponsoft.co.jp/solution/bellemax/). The datasets regarding confirmed cases of COVID-19 are public.

## Code availability

The effective reproduction number time series was calculated using the R package “EpiEstim” in R 3.6.1. Due to the privacy policies associated with the spatiotemporal population data and other private data, the code for the analysis is not available publicly, but is available from the corresponding author upon reasonable request.
