# Supplementary material for: Revealing spatiotemporal variations in areas potentially linked to COVID-19 spread using fine-grained population data
Source: Sci Rep. 2025 Jul 2;15:22636. doi: 10.1038/s41598-025-06658-7 (PMC12215455; doi:10.1038/s41598-025-06658-7)
Supplement: Supplementary file 1 — Supplementary Information. [file 41598_2025_6658_MOESM1_ESM.pdf]

Supplementary information for *Revealing spatiotemporal variations in areas potentially linked to COVID-19 spread using fine-grained population data*

This document contains supplementary figures and tables for the main text.

List of Figures

S1 The number of confirmed cases in Tokyo. The grey bars represent the number of confirmed cases per day. The red curve represents the 7-day moving average of the number of confirmed cases. Periods of the waves are depicted by shading. 3

S2 Total number of reported cases per ward during a given period. 4

S3 Periods selected based on CV of R_t. The period for each ward is denoted by blue bars, and accounts for the reporting delays. The vertical grey lines show the beginning and end of each wave of the pandemic. See also Table S3 for details. 5

S4 Effective reproduction number and confirmed cases of each ward. The graphs are cut off on 20 October 2021 to avoid jumps in Rt during October 2021 for visualization. Every quantity is based on the reporting date. 6

S5 Ratio of population with two doses of vaccines against COVID-19 to the population, including age stratification. 7

S6 A wide version of the correlation map for Setagaya Ward residents in the third wave in Fig. 2. 8

S7 Potential areas of concern for Adachi Ward residents during different waves. Adachi Ward is shown in purple. The colour of each cell represents the time of day for which the population time series had the highest correlation with R_t. Railways are shown as grey curves, of which the Yamanote Line is highlighted in black. 9

S8 Comparison of top 300 cells regarding (**a**) our risk metric and (**b**) population ranking. The points are facilities with infections. 10

S9 The ratio of the number of POIs in cells of potential concern for Setagaya Ward residents to the total number of POIs in each category. 11

S10 The number of POIs in cells of potential concern for Adachi Ward residents. 12

S11 The number of POIs in cells of potential concern for Edogawa Ward residents. 12

S12 The number of POIs in cells of potential concern for Nerima Ward residents. 13

S13 The number of POIs in cells of potential concern for Ota Ward residents. 13

List of Tables

S1 Period of waves of the pandemic in Tokyo. The periods were determined based on the reporting day. 2
S2 Periods of States of Emergency. Each measure includes: (i) a request for residents to refrain from going out, (ii) a request for shortened business hours for restaurants and similar establishments, (iii) a request for closure or reduced operating hours of event-related and leisure facilities, and (iv) restrictions on the scale of event hosting. 5
S3 Details of the periods selected based on CV of R_t. 5
S4 List of excluded words in Japanese. 7

Data and statistics regarding COVID-19

Periods of the pandemic waves

See Table S1 and Fig. S1.

Table S1: Period of waves of the pandemic in Tokyo. The periods were determined based on the reporting day.

Wave	Beginning	End
1st	10 March 2020	19 May 2020
2nd	20 May 2020	4 September 2020
3rd	2 November 2020	23 February 2021
4th	5 March 2021	12 June 2021
5th	16 June 2021	31 October 2021

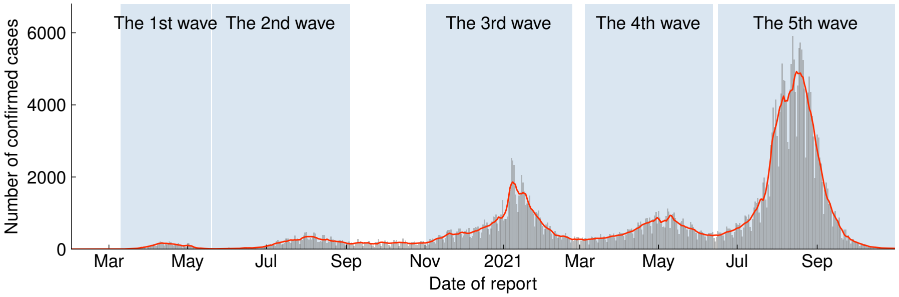

Fig. S1: The number of confirmed cases in Tokyo. The grey bars represent the number of confirmed cases per day. The red curve represents the 7-day moving average of the number of confirmed cases. Periods of the waves are depicted by shading.

Ranking of wards on total reported cases

See Fig. S2.

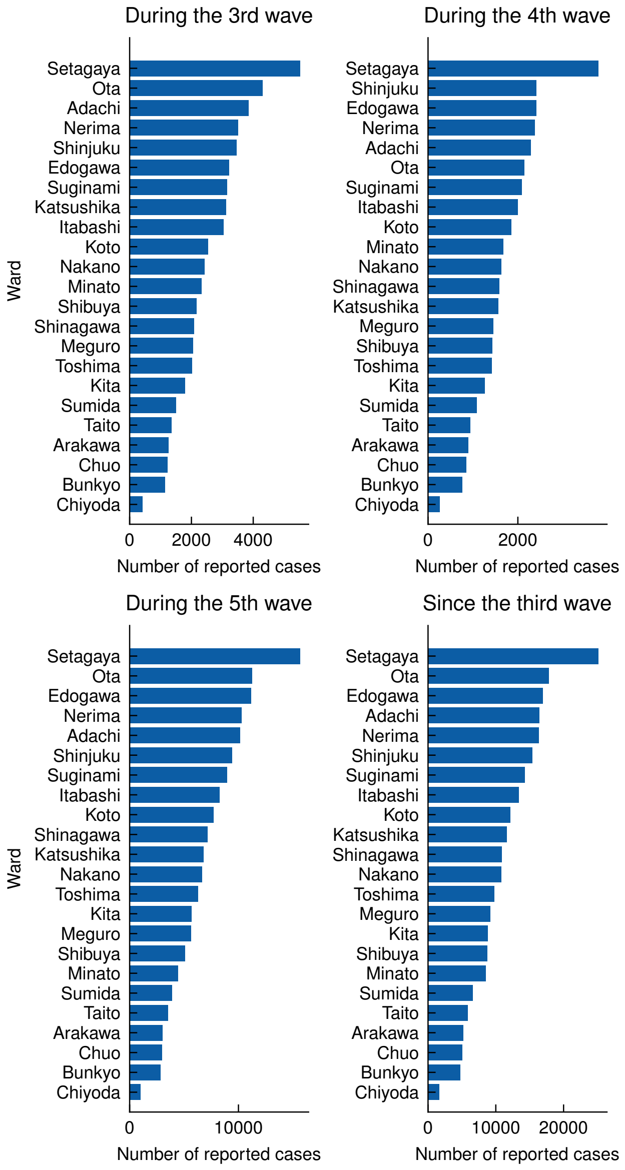

Fig. S2: Total number of reported cases per ward during a given period.

Periods of states of emergency

See Table S2.

Table S2: Periods of States of Emergency. Each measure includes: (i) a request for residents to refrain from going out, (ii) a request for shortened business hours for restaurants and similar establishments, (iii) a request for closure or reduced operating hours of event-related and leisure facilities, and (iv) restrictions on the scale of event hosting.

Measure type	Beginning	End
State of emergency	7 April 2020	25 May 2020
State of emergency	8 January 2021	21 March 2021
Semi-state of emergency	12 April 2021	24 April 2021
State of emergency	25 April 2021	20 June 2021
Semi-state of emergency	21 June 2021	11 July 2021
State of emergency	12 July 2021	30 September 2021

Selected periods for each ward

See Fig. S3 and Table S3.

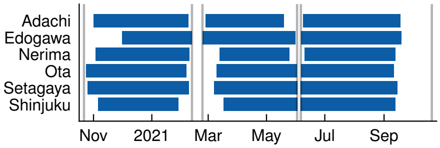

Fig. S3: Periods selected based on CV of R_t. The period for each ward is denoted by blue bars, and accounts for the reporting delays. The vertical grey lines show the beginning and end of each wave of the pandemic. See also Table S3 for details.

Table S3: Details of the periods selected based on CV of R_t.

Ward	For the 3rd wave	For the 4th wave	For the 5th wave
Adachi	2020-11-01 to 2021-02-08	2021-02-26 to 2021-05-19	2021-06-08 to 2021-09-18
Edogawa	2020-12-01 to 2021-02-12	2021-02-23 to 2021-05-31	2021-06-06 to 2021-09-19
Nerima	2020-11-03 to 2021-02-09	2021-03-13 to 2021-05-25	2021-06-10 to 2021-09-13
Ota	2020-10-24 to 2021-02-06	2021-03-10 to 2021-06-02	2021-06-06 to 2021-09-11
Setagaya	2020-10-26 to 2021-02-09	2021-03-07 to 2021-06-02	2021-06-06 to 2021-09-15
Shinjuku	2020-11-06 to 2021-01-29	2021-03-17 to 2021-06-02	2021-06-06 to 2021-09-13

Estimated effective reproduction number

See Fig. S4.

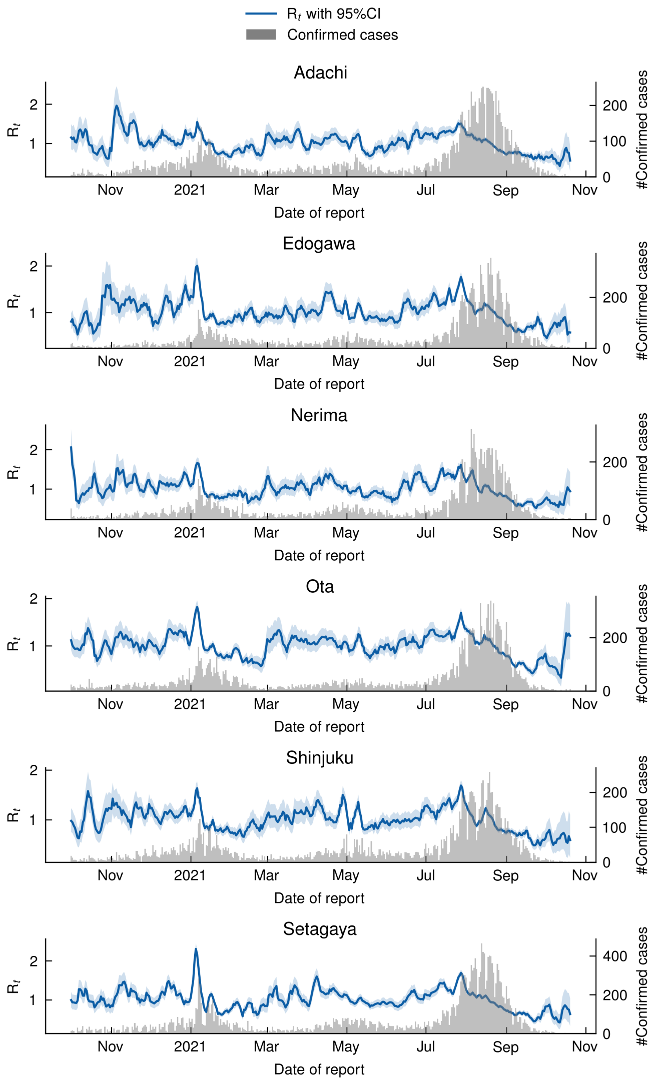

Fig. S4: Effective reproduction number and confirmed cases of each ward. The graphs are cut off on 20 October 2021 to avoid jumps in *Rt* during October 2021 for visualization. Every quantity is based on the reporting date.

Excluded facilities

See Table S4.

Table S4: List of excluded words in Japanese.

Category	Japanese
hospital	病院，医院，クリニック，診療
nursing home	介護，老人，デイサービス
school	学校
kindergarten	保育，幼稚園

Vaccination statistics of Tokyo

See Fig. S5.

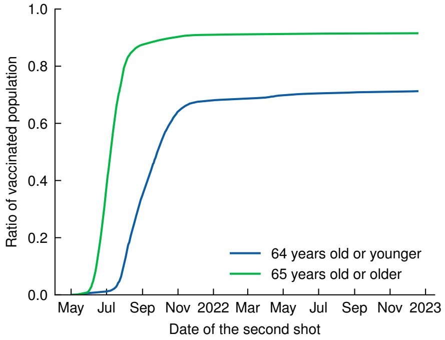

Fig. S5: Ratio of population with two doses of vaccines against COVID-19 to the population, including age stratification.

Maps of potential areas of concern

See Figs. S6 and S7.

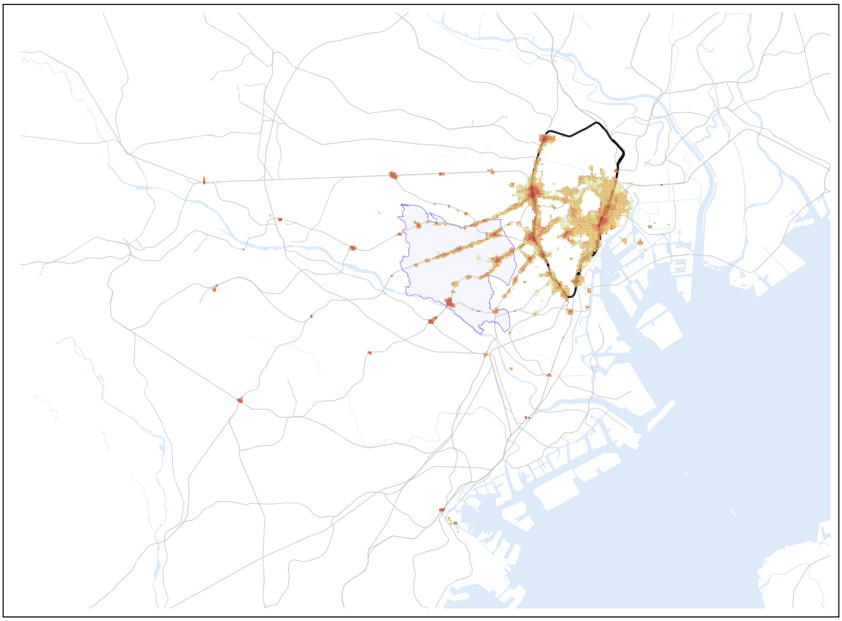

Fig. S6: A wide version of the correlation map for Setagaya Ward residents in the third wave in Fig. 2.

Risk Calculation

Parameter determination

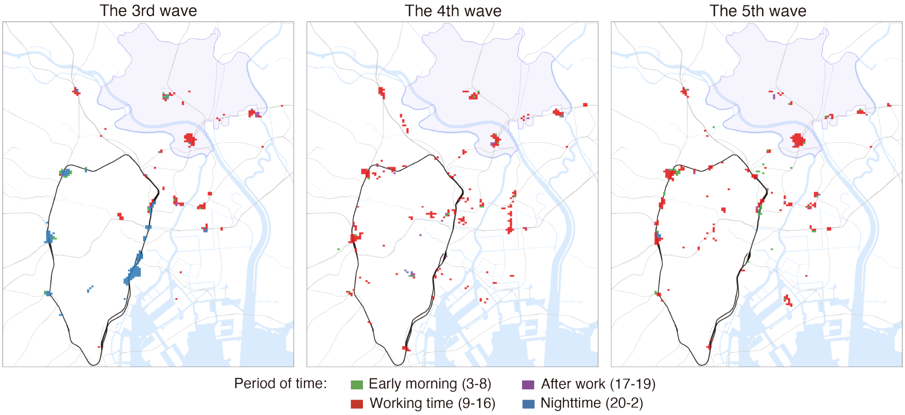

Fig. S7: Potential areas of concern for Adachi Ward residents during different waves. Adachi Ward is shown in purple. The colour of each cell represents the time of day for which the population time series had the highest correlation with R_t. Railways are shown as grey curves, of which the Yamanote Line is highlighted in black.

a

b

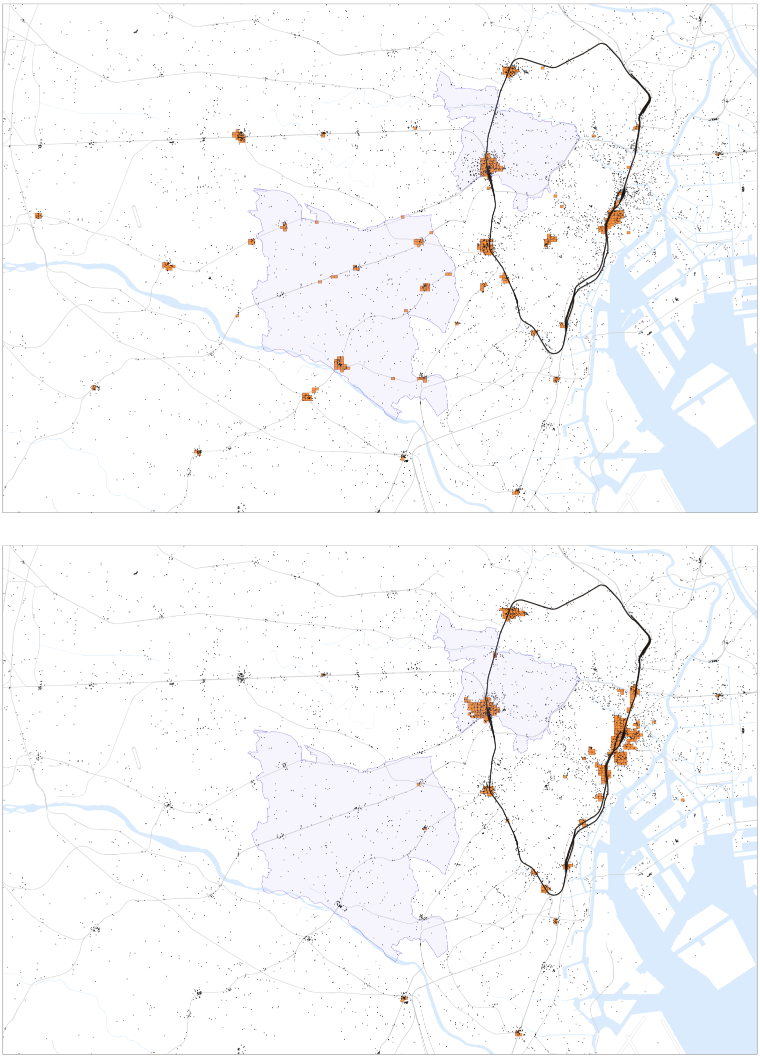

Fig. S8: Comparison of top 300 cells regarding (**a**) our risk metric and (**b**) population ranking. The points are facilities with infections.

POIs analysis

See Figs. S9 to S13.

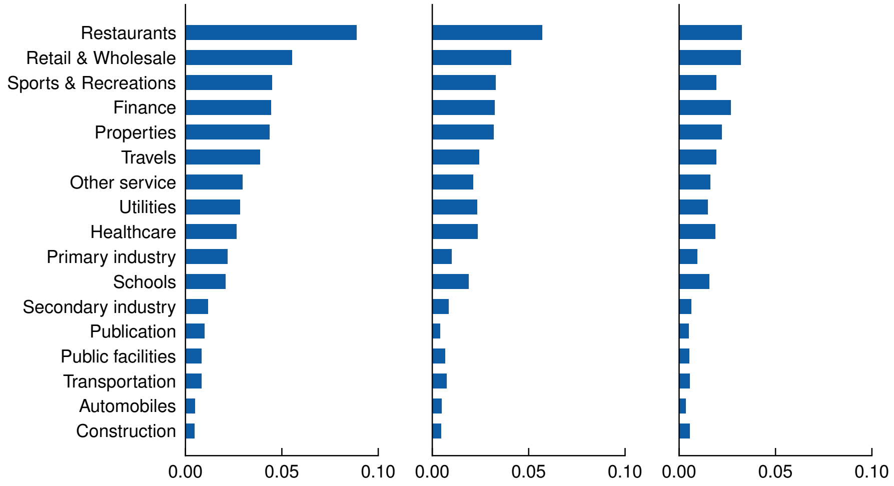

Fig. S9: The ratio of the number of POIs in cells of potential concern for Setagaya Ward residents to the total number of POIs in each category.

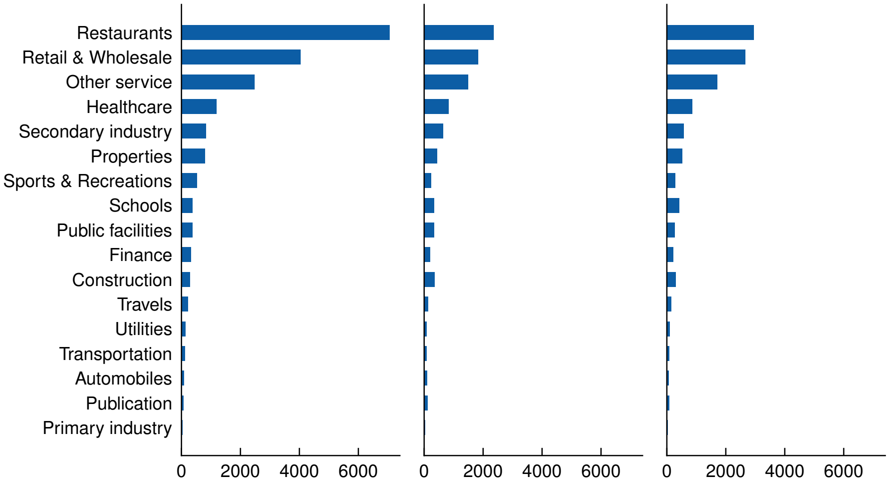

Fig. S10: The number of POIs in cells of potential concern for Adachi Ward residents.

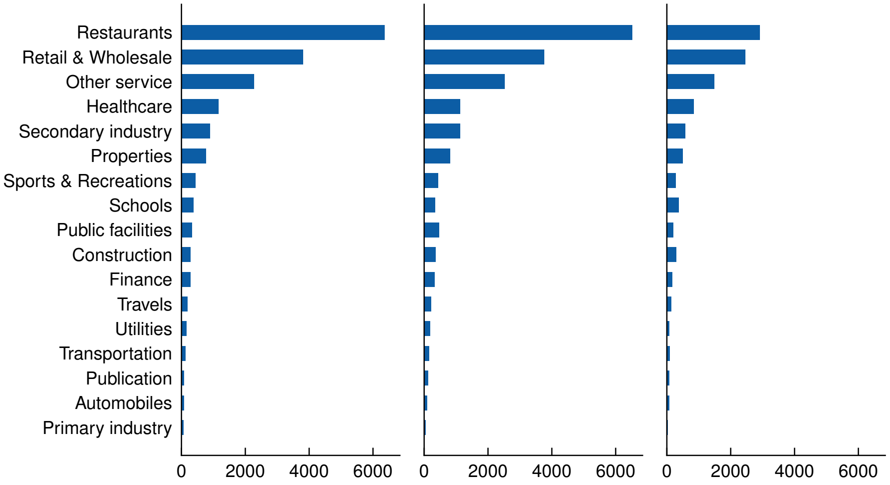

Fig. S11: The number of POIs in cells of potential concern for Edogawa Ward residents.

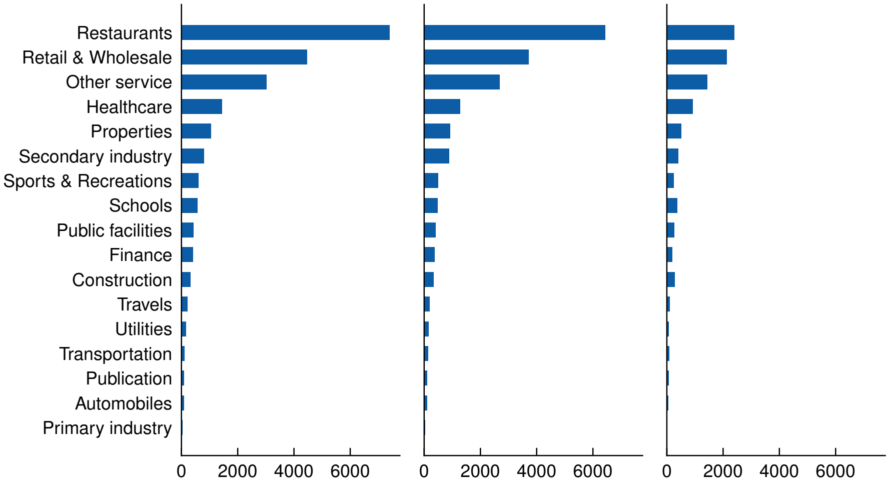

Fig. S12: The number of POIs in cells of potential concern for Nerima Ward residents.

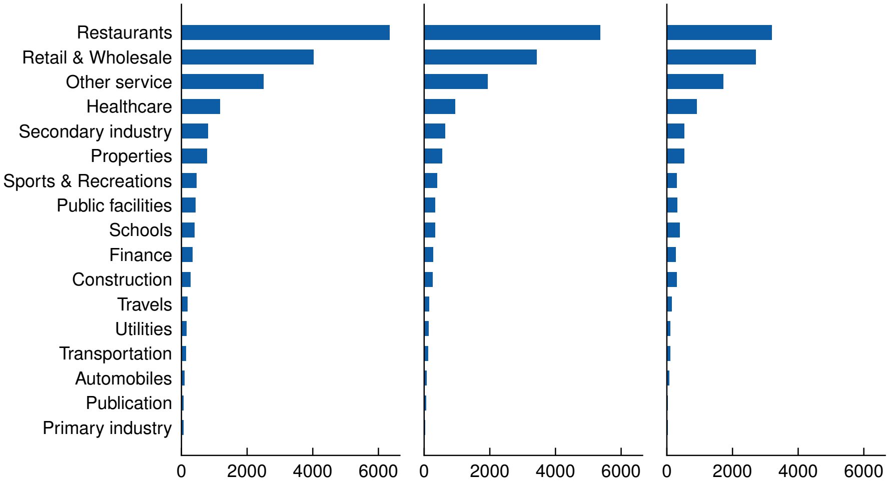

Fig. S13: The number of POIs in cells of potential concern for Ota Ward residents.
